# Supplementary material for: Microfluidic manipulation by spiral hollow-fibre actuators
Source: Nat Commun. 2022 Mar 14;13:1331. doi: 10.1038/s41467-022-29088-9 (PMC8921237; doi:10.1038/s41467-022-29088-9)
Supplement: Supplementary file 1 — Supplementary Information [file 41467_2022_29088_MOESM1_ESM.pdf]

Supplementary information for

Microfluidic Manipulation by Spiral Hollow-Fiber Actuators

Sitong Li[1], Rui Zhang[2], Guanghao Zhang[1], Luyizheng Shuai[1], Wang Chang[1], Xiaoyu Hu[1], Min Zou[1], Xiang Zhou[1], Baigang An[3], Dong Qian[2], Zunfeng Liu[1]*

[1]State Key Laboratory of Medicinal Chemical Biology, College of Chemistry and College of Pharmacy, Key Laboratory of Functional Polymer Materials, Frontiers Science Center for New Organic Matter, Nankai University, Tianjin 300071, China
[2]Department of Mechanical Engineering, University of Texas at Dallas, Richardson, TX 75080, USA
[3]School of Chemical Engineering, University of Science and Technology Liaoning, Anshan 114051, China
E-mail: liuzunfeng@nankai.edu.cn

This PDF file includes:

Supplementary Note 1. Methods (Pages S2–S3)
Supplementary Note 2. Thermomechanical modeling (Pages S3–S7)
Supplementary Note 3. Optimum hollow fiber geometry analysis (Pages S7–S8)
Supplementary Figures 1 to 12 (Pages S9–S15)
Supplementary Tables 1 to 3 (Pages S16-17)
References (Page S18)

Supplementary Note 1. Methods

1.1 Characterizations

Linear coefficients of thermal expansion (CTE) of PEHF were measured from 0 to 100 °C at a heating rate of 3 °C min^{-1} using a thermomechanical analyzer (TMA, Q400 system, TA instruments). Because the thermal expansion of employed polyethylene hollow fibre is anisotropic[1], the axial and the radial coefficients of thermal expansion were tested individually. For each experiment, three heating scans and two cooling scans were conducted. The linear CTE along each direction was calculated based on the thermomechanical analysis results.

Two-dimensional wide-angle X-ray scattering (2D WAXS) experiments were performed at a sample-to-detector distance of 100 mm using CuKα radiation ($\lambda = 1.54$ Å) by deploying a D8 Discover system (Bruker, Germany) equipped with a 2D HI-Star detector and a 1.6 kW generator. Parallel PEHF were assembled, using a video microscope to ensure that the X-ray beam irradiated the centers of the fibres. The image acquisition time was 600 s. One-dimensional wide-angle X-ray scattering (1D WAXS) experiments were performed using CuKα radiation and a 2θ scanning rate of 5° min^{-1} on a Rigaku Smart Lab system (Japan) equipped with a 3.0 kW generator.

1.2 Calculation of the degree of orientation of crystals using WAXS analysis

Despite inserting twists into a polymer fibre, the highly oriented semicrystalline form is also the key to provide a fibre the actuation capability. The Herman's orientation parameter (*f*) is employed to represent the polymer chain alignment of the fibre, where $f = 1$ means that the polymer chains orient along the fibre axis direction completely; $f = -0.5$ means that the polymer chains orient vertically to the fibre axis direction completely. For a non-oriented sample, f equals to 0.

The Herman's orientation parameters (f_a, f_b, and f_c) for the a-axis, b-axis, and c-axis, for a unit cell of polyethylene crystallite with orthorhombic symmetry, were calculated as 0.12, -0.30, and 0.18, respectively, according to the literatures.[2-3] These values provide the evidences that the fibres are in highly oriented semicrystalline

form.

The calculation details are as follows.

The Herman's orientation parameter (f) is defined as:

$$f = \frac{3\langle \cos^2 \Phi \rangle - 1}{2} \quad (1.1),$$

where $\langle \cos^2 \Phi \rangle$ is an orientation factor defined as:

$$\langle \cos^2 \Phi \rangle = \frac{\int_0^{\pi} I_{\Phi} \cos^2 \Phi \sin \Phi \mathrm{d}\Phi}{\int_0^{\pi} I_{\Phi} \sin \Phi \mathrm{d}\Phi} \quad (1.2),$$

where I_{Φ} represents the diffraction intensity. The azimuthal curve obtained by integration of diffraction intensity in the 2D WAXS pattern is used in Eq. (1.2) to calculate $\cos^2 \Phi$ by the numerical integration process. Then the values of $\cos^2 \Phi$ are used in Eq. (1.1) to calculate the orientation function value f of the semicrystalline polymers.

For polyethylene with orthorhombic symmetry of the unit cell, the a-axis (f_{a}) and b-axis (f_{b}) orientation parameters were calculated from the intense reflections of (200) and (020) planes based on the relation:

$$\langle \cos^2 \Phi_{110} \rangle = 0.692\langle \cos^2 \Phi_{020} \rangle + 0.308\langle \cos^2 \Phi_{200} \rangle \quad (1.3),$$

where $\langle \cos^2 \Phi_{110} \rangle$ and $\langle \cos^2 \Phi_{200} \rangle$ are obtained from the azimuthal intensity measurements on the (110) and (200) reflections, respectively. The parameter f_{c} is estimated based on the orthogonal symmetry relationship: $f_{\mathrm{a}} + f_{\mathrm{b}} + f_{\mathrm{c}} = 0$.[4]

The detailed calculations for $f_a, f_b,$ and f_c are as follows. First, an azimuthal curve was obtained by integration of diffraction intensity in the 2D WAXS pattern over the azimuthal angle using the software of the GADDS (general area detector diffraction system) for diffraction angles ($2\theta_{\mathrm{d}}$) of 21.6° (110 plane) and 24° (200 plane), as shown in Supplementary Fig. 1c. Two identical peaks were observed in the azimuthal curve (Supplementary Fig.1d) because of the symmetric diffraction pattern in 2D WAXS. The $f_a, f_b,$ and f_c of the PEHF are respectively 0.12, -0.30, and 0.18, which indicate that the fibres are in highly oriented semicrystalline form.

Supplementary Note 2. Thermomechanical modeling of hollow fibre actuator

1.1 Transient heat conduction analysis

The temperature change of the hollow fibre actuator is contributed by the forced convection of the internal flowing water as well as the natural convection of the ambient (external) air. To study this transient heat conduction problem, we establish a two-step model. First, we model the radial heat conduction at an arbitrary cross-section of the hollow fibre actuator. Second, we build a simple axial heat conduction model based on the assumption that heat conduction rate along the hollow fibre axis is much slower compared to the water flow rate and the rate of heat conduction in the radial direction. With this two-step model, we determine the hollow fibre actuator temperature as a function of radial and axial coordinates and time.

The DSC results showed that there was no observable phase transition in the PEHF for the investigated temperature range (Supplementary Fig. 1b), we assume that there is no contribution of the phase transition to the volume change and the heat transfer contributed to the volume expansion.

First, the governing equation of the transient heat conduction in radial direction at an arbitrary cross-section of the hollow fibre is given by

$$\frac{\partial^2\theta}{\partial r^2}+\frac{1}{r}\frac{\partial\theta}{\partial r}=\frac{1}{\alpha}\frac{\partial\theta}{\partial t} \quad \text{in } R_i \le r \le R_o, \tag{2.1}$$

where θ (r, t) is the temperature, R_i and R_o are the inner and outer radius of the hollow fibre, respectively. Thermal diffusivity α is defined by

$$\alpha=\frac{k}{\rho c_p}, \tag{2.2}$$

in which k is the thermal conductivity, ρ is the mass density, c_p is the specific heat capacity[5-6]. For PE, we have k = 0.33 W $(\text{m}\cdot\text{K})^{-1}$,[7] ρ=920 kg m^{-3},[8] c_p = 2300 J $(\text{kg}\cdot\text{K})^{-1}$ [8].

The convective boundary conditions at the interior and exterior surfaces of the hollow fibre are given by

$$k\frac{\partial\theta}{\partial r}\bigg|_{r=R_i} = h_w\left(\theta\big|_{r=R_i} - \theta_w\right), \tag{2.3}$$

$$-k\frac{\partial\theta}{\partial r}\bigg|_{r=R_o} = h_a\left(\theta\big|_{r=R_o} - \theta_a\right), \tag{2.4}$$

where h_w and h_a are the heat transfer coefficients of water and air, respectively. Similarly, θ_w and θ_a are respectively the temperatures of water and air. The heat transfer coefficient of water depends on many factors such as hollow fibre internal geometry, surface roughness, water physical properties as well as the flow state (laminar or turbulent), *etc*. In this work, we employ h_w = 2000 W $(m^2{\cdot}K)^{-1}$, which provides the best fit of theoretical predictions to the experimental measurements.

The initial condition is

$$\theta(t=0) = \theta_0, \tag{2.5}$$

where θ_0 is the initial temperature of the hollow fibre actuator.

The above 1D radial transient heat conduction equations can be solved either analytically using methods of separation of variables and superposition, which are well documented in ref. 10, or using numerical methods such as finite difference or finite element. In this work, we solved this 1D partial differential equation (PDE) using the PDE solver provided by MATLAB*.

Next, we assume that the axial heat conduction rate is much lower compared to the water flow rate. As such, the heat transfer in the axial direction is mainly contributed by the flowing water and the radial conduction. Therefore, by substituting water temperature as a function of axial coordinate and time, i.e. $\theta_w = \theta_w(x,t)$, in which x is the axial coordinate, into the above radial heat conduction equations, we can solve the hollow fibre temperature distribution in the axial direction.

Water temperature θ_w (x, t) depends on a few factors, such as initial water temperature, water flow rate, heat loss due to transfer to hollow fibre actuator and

---

*https://www.mathworks.com/help/matlab/math/partial-differential-equations.html

eventually to the ambient air, *etc*. For simplicity, we assume that the heat loss is relatively low when the flow rate is high. Therefore, we can write the water temperature as

$$\theta_w(x,t) = \theta_{w0} H(vt - x), \tag{2.6}$$

where θ_{w0} is the initial water temperature, v is the water flowrate and $H(\cdot)$ is the Heaviside step function.

2.2 Torsional actuation modeling

Torsional actuation mechanism of the twisted polymer fibres was studied in ref. 10 and 11. The hollow fibre length l, the hollow fibre outer diameter d, and the helically oriented polymer chain length λ are related through

$$\lambda^2 = \left[(\pi d T)^2 + 1\right] l^2, \tag{2.7}$$

where T is the twist density.

Upon heating, polymer chains will expand in the radial direction and contract in the axial direction, which can be accommodated by the changes in the hollow fibre length, the diameter and the twist density. In ref. 10, the change of hollow fibre length is considered small and the following equation for untwisting can be obtained by

$$\Delta T = \left(\frac{\Delta\lambda}{\lambda}\frac{1}{\cos^2\alpha_f} - \frac{\Delta d}{d}\right)T, \tag{2.8}$$

where bias angle $\alpha_f = \tan^{-1}(\pi d T)$ [12].

Ref. 11 further assumed that λ is also constant. Therefore, the untwist ΔT is linearly proportional to the change in the hollow fibre diameter, i.e.

$$\Delta T = -\frac{\Delta d}{d} T, \tag{2.9}$$

In this work, we employ Eq. (2.8) for torsional actuation modeling. It can be rewritten as

$$\Delta T = \left(\alpha_\lambda \frac{1}{\cos^2\alpha_f} - \alpha_d\right)\Delta\theta \cdot T, \tag{2.10}$$

where α_λ and α_d are the coefficients of thermal expansion of polymer chain in the axial direction and that of the hollow fibre in the radial direction, respectively. The $\Delta\theta$ is the temperature change obtained from the previous transient heat conduction analysis. In this work, the average exterior hollow fibre temperature change is employed as the actuation temperature $\Delta\theta$. Axial and radial coefficients of thermal expansion of PEHFs were employed as $\alpha_\lambda = -(5.3 \pm 0.4) \times 10^{-4}$ K^{-1} and $\alpha_d = (5.2 \pm 0.7) \times 10^{-4}$ K^{-1}, which were obtained from thermomechanical analysis (TMA).

2.3 Tensile actuation modeling

The tensile actuation mechanism of the twisted, mandrel-coiled polymer fibre is well documented in ref. 10, which is adopted in this work. The equation for tensile actuation is

$$\frac{\Delta L}{L} = \frac{l^2}{NL}\Delta T \quad , \qquad (2.11)$$

where L is the coil length, l is the twisted hollow fibre length, N is the number of coil turns and ΔT is the amount of untwisting that can be obtained from Eq. (2.10).

Supplementary Note 3. The analysis for the optimum hollow fibre geometry

To provide an estimation of the optimum configuration of the hollow fibre, we assume steady heat solution and constant temperature due to the small thickness of the hollow fibre. Based on the linear elastic solution for a thin-walled hollow fibre under pure torsion[13], the generated torque M is related to the twist through

$$M = 2\pi GJT, \qquad (3.1)$$

in which G is shear modulus, J is the polar second moment of area. For the hollow fibre section $J = \frac{\pi}{2}\left(r_o^4 - r_i^4\right)$ where r_i and r_o are the inner and outer radius of the hollow fibre, respectively.

Applying Eq. (3.1) to the hollow fibre configurations before and after the

torsional actuation provides the torque release ΔM:

$$\Delta M = \pi G A_0\left(r_i^2 + r_o^2\right)\left[(1+\alpha_d \Delta\theta)^4 (T+\Delta T) - T\right], \tag{3.2}$$

in which A_0 is the cross-section area of the hollow fibre.

We now introduce a simplified version of torsional actuation based on Ref. 10 :

$$\Delta T = \left(\frac{d_0}{d} - 1\right) T. \tag{3.3}$$

Substituting Eq. (3.3) into (3.2) gives

$$\Delta M = \pi G A_0\left(r_i^2 + r_o^2\right)\left[(1+\alpha_d \Delta\theta)^3 - 1\right] T. \tag{3.4}$$

Based on Eq. (3.4), we conclude that the optimum geometry for maximizing the torque output is to maximize $\left(r_i^2 + r_o^2\right)$, assuming that the cross-section area A_0 (or equivalently the material consumption) is constant. This means that the material should be distributed as far away as possible from the center of the cross-section, which justifies the use of hollow fibre over solid cross-sections. It should be noted that the above theoretically derived optimum geometry is subjected to the constraint that the hollow fibre will not buckle under the applied torque. Thus, in practice a hollow fibre with finite thickness should be used for the proposed actuation application.

Supplementary Figures

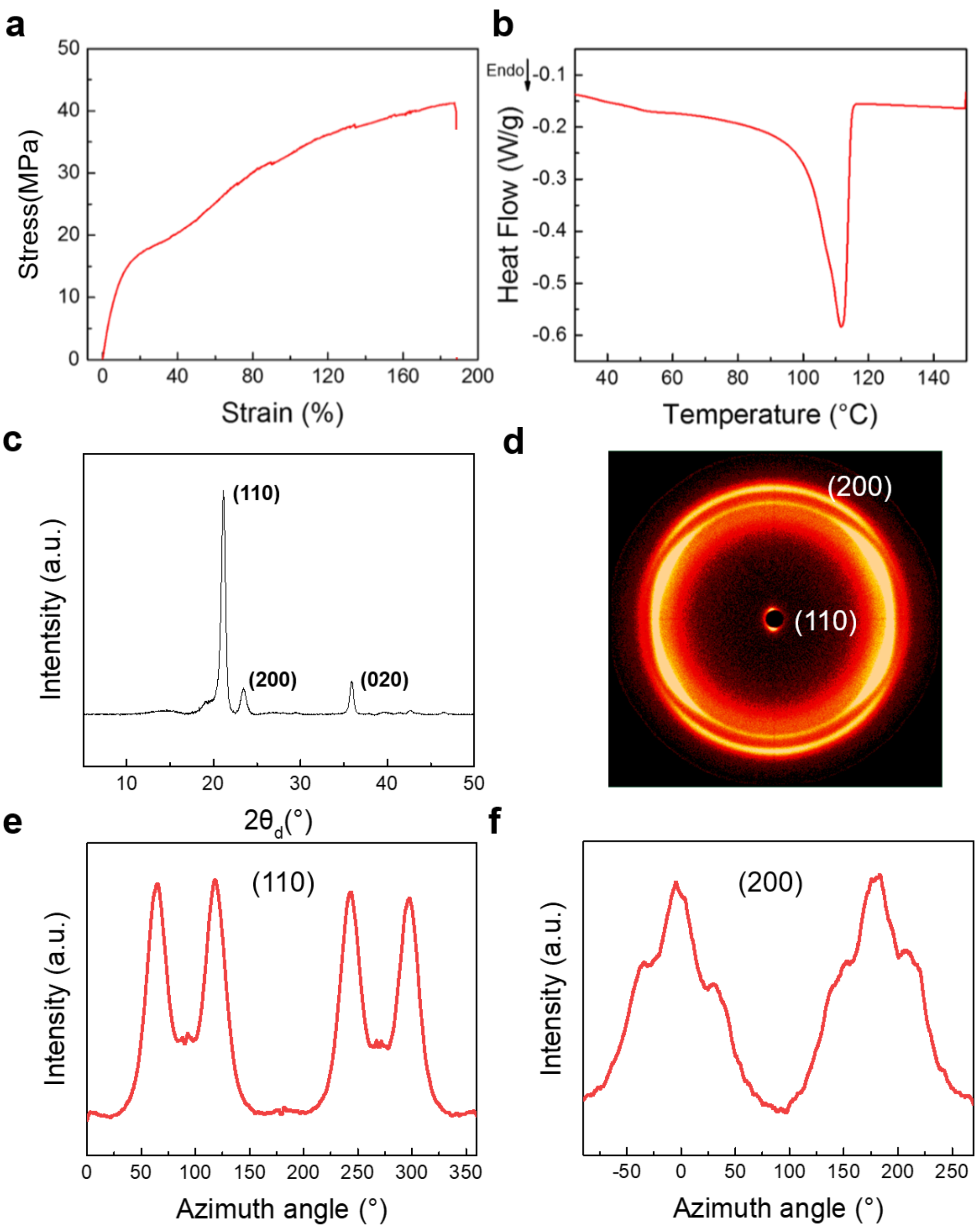

Supplementary Figure 1. (a) The stress-strain curve of the $PEHF_{580\text{-}990}$. The gauge length was 34 mm, and the extension rate was 20 mm min^{-1}. (b) Differential scanning calorimetry (DSC) curve of the $PEHF_{580\text{-}990}$ with a heating rate of 5°C/min in N_2 atmosphere. The melting enthalpy (H_m=126.4 J g^{-1}) was obtained by integration of the endothermic melting peak. By considering that the melting enthalpy of the 100% crystalline polyethylene was H_c=287.3 J g^{-1} [14], we can obtain the crystallinity of low-density polyethylene (χ_c) from the equation $\chi_c=H_m/H_c$=44%. (c) The intensity of diffraction peaks versus Bragg angle (2θ) in WAXS for $PEHF_{580\text{-}990}$. (d) 2D WAXS diffraction pattern for $PEHF_{580\text{-}990}$ showing anisotropic structure. (e, f) The azimuthal curves of (110) and (200) planes in $PEHF_{580\text{-}990}$ were obtained from (d).

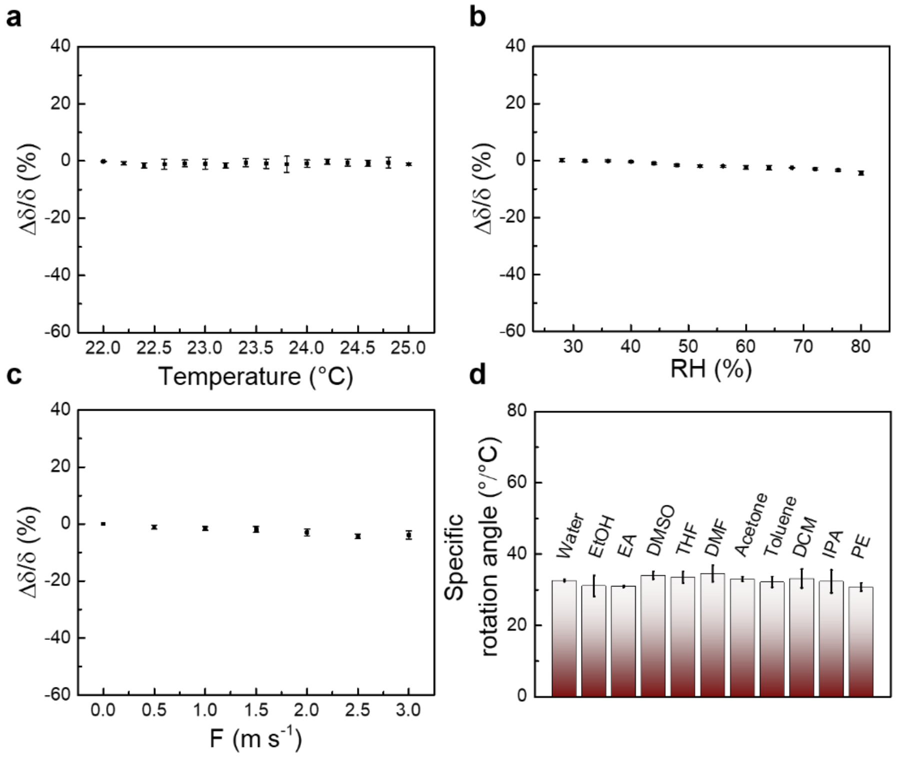

Supplementary Figure 2. The torsional actuation of the $PEHF_{580-990}$ actuator by flowing hot water at 1.72 g s^{-1} at different actuation conditions: (a) at different environment temperatures, (b) at different environmental relative humidity, and (c) at different wind speed. (d) The torsional actuation of the $PEHF_{580-990}$ actuator by flowing different types of 40 °C liquid at a flow rate of 1.72 g s^{-1}. The δ and Δδ are the rotation angle during actuation and its change. If not specified, the room temperature is 25 °C, the environmental relative humidity is 40%, and the water flow rate is 1.72 g s^{-1}. If not specified, error bars in this figure and in the following figures indicate standard deviations. EtOH: ethanol; EA: ethyl acetate; DMSO: dimethyl sulfoxide; THF: tetrahydrofuran; DMF: N, N-dimethylformamide; DCM: dichloromethane; IPA: iso-propanol; PE: petroleum ether.

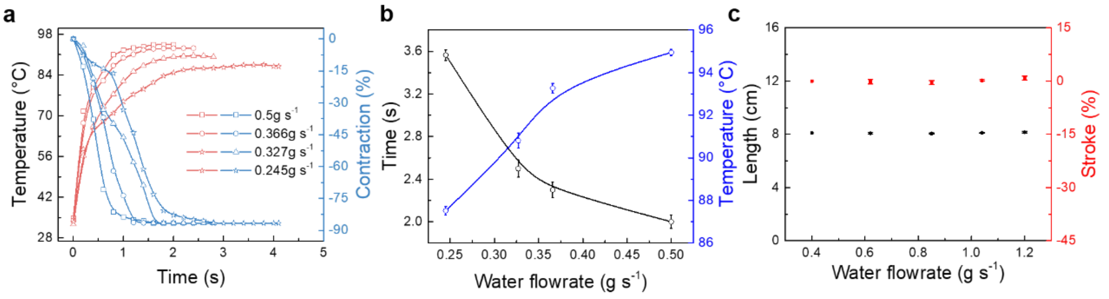

Supplementary Figure 3. (a) Contraction and hollow fibre surface temperature as a function of time for a homochiral $PEHF_{580-990}$ actuator by flowing 95 °C water with different flow rates. (b) Response time and hollow fibre surface temperature as a function of water flow rate for a homochiral $PEHF_{580-990}$ actuator by flowing 95 °C water. (c) Coil length and actuation stroke of the homochiral $PEHF_{580-990}$ actuator by

flowing 25 °C water at different flow rates. The spring index was 4.0, and the inserted twist density was 200 turns m^{-1}.

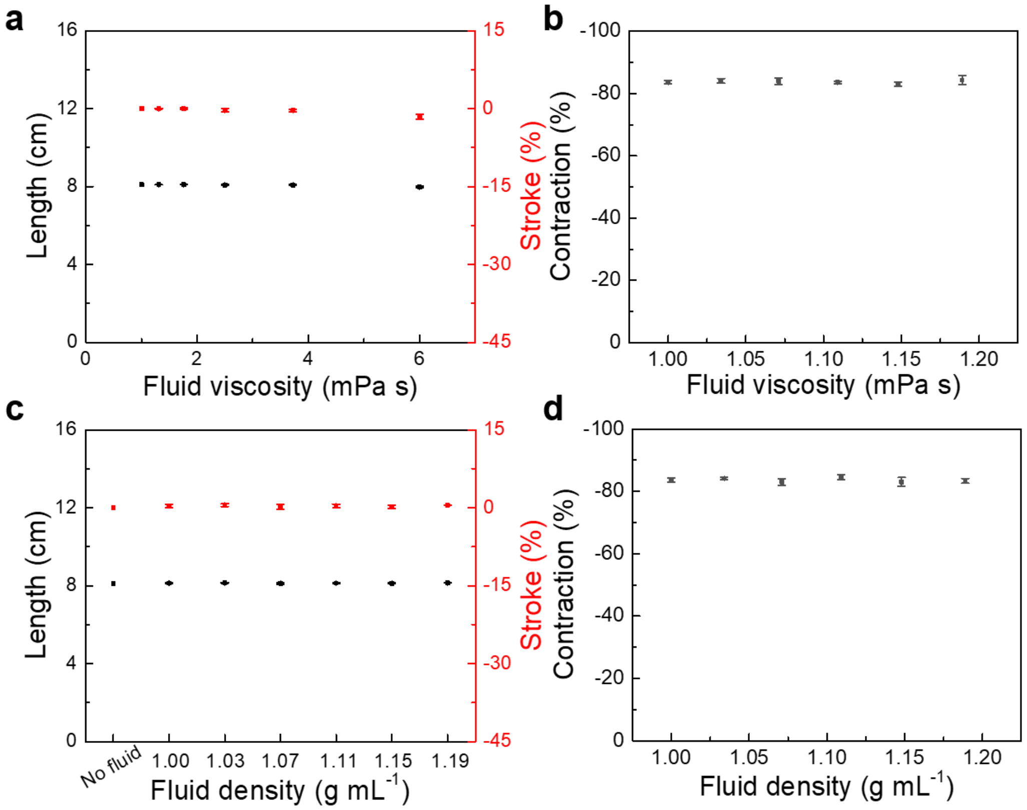

Supplementary Figure 4. (a) The initial coil length and actuation stroke of the homochiral $PEHF_{580-990}$ actuator by flowing 25 °C glycerol/water solution with different viscosity. (b) Actuation stroke of the $PEHF_{580-990}$ actuator by flowing 80 °C glycerol/water solution with different viscosity. (c) The initial coil length and actuation stroke of the $PEHF_{580-990}$ actuator by flowing 25 °C aqueous NaCl solution with different densities. (d) Actuation stroke of the $PEHF_{580-990}$ actuator by flowing 80 °C aqueous NaCl solution with different densities. The spring index was 4.0, and the twist density was 200 turns m^{-1}.

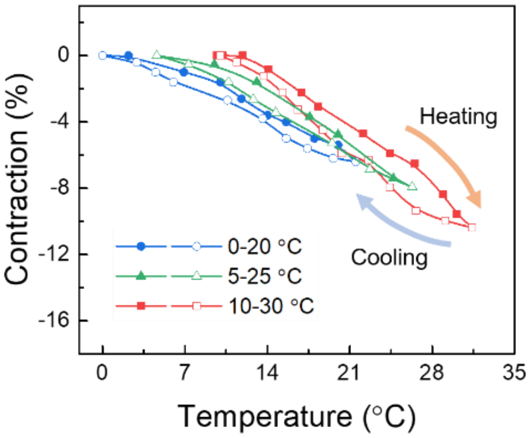

Supplementary Figure 5. The contraction as a function of temperature for $PEHF_{580-990}$ actuator driven by consecutively flowing hot water and cold water with a temperature range. The spring index was 4.0, the inserted twist density was 200 turns

m^{-1}, and the flow rate was 1.72 g s^{-1}. The solid symbols indicate the heating process, and the open symbols indicate the cooling process.

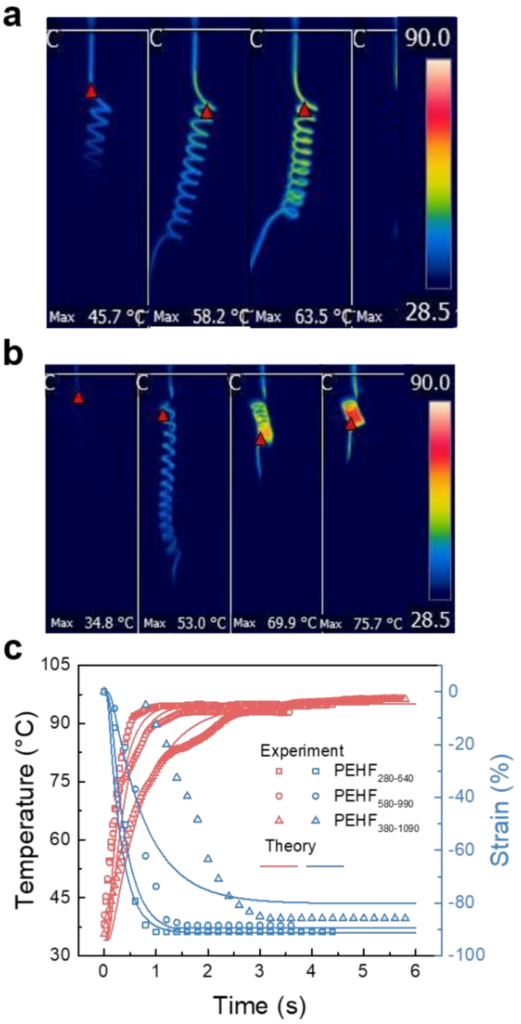

Supplementary Figure 6. (a, b) Serial infrared images of the homochiral PEHF$_{380\text{-}1090}$ (a) and PEHF$_{280\text{-}640}$ (b) actuators by flowing 95 °C water at different time (0 s, 0.2 s, 0.4 s, and 0.6 s). (c) Experimental results and theoretical analysis of the actuation strain and surface temperature of the PEHF actuators as a function of time.

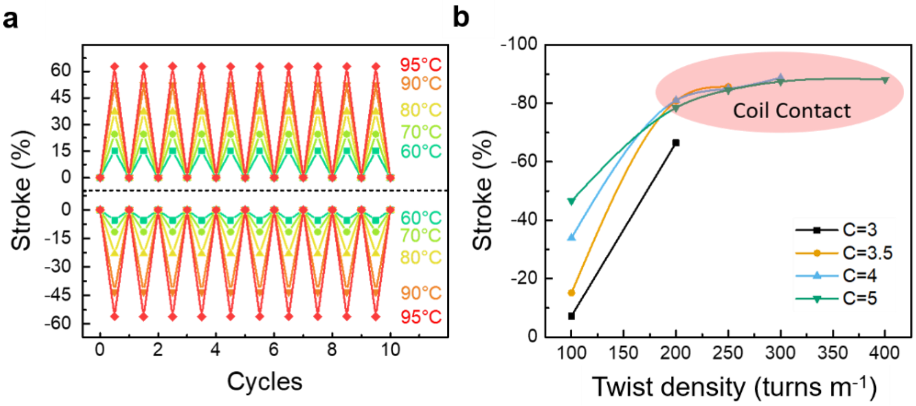

Supplementary Figure 7. (a) Maximum actuation stroke for ten consecutive heating/cooling cycles for heterochiral and homochiral load-free PEHF$_{580\text{-}990}$ actuators driven by alternatively flowing hot water and 25 °C water. (b) Maximum actuation

stroke as a function of twist density for load-free PEHF$_{580\text{-}990}$ homochiral actuators with different spring indexes driven by 80 °C flowing water.

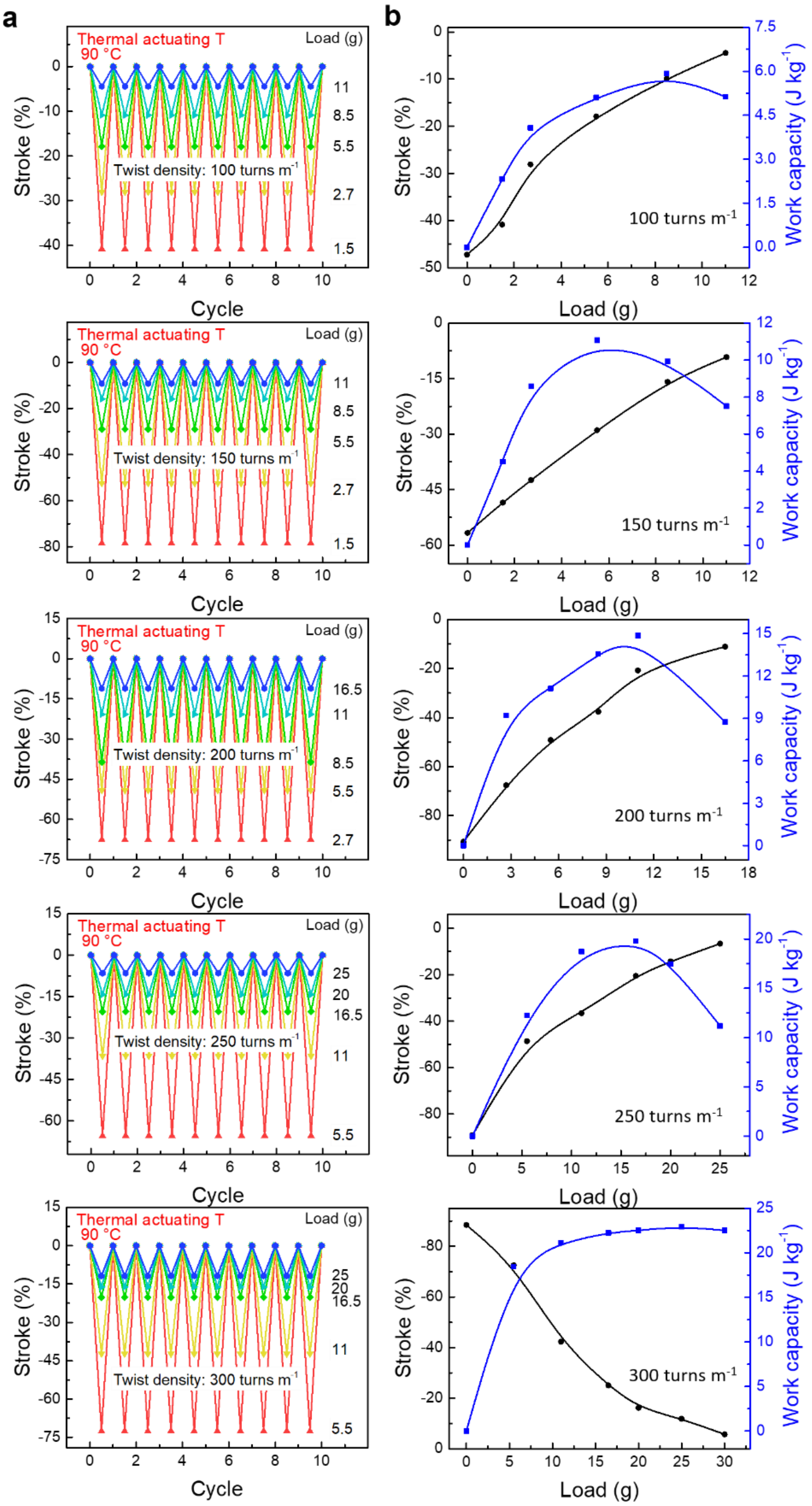

Supplementary Figure 8. (a) Maximum contraction at different mass of the load for ten consecutive heating/cooling cycles of homochiral PEHF$_{580\text{-}990}$ actuators with a spring index of 4.0 and an inserted twist of 100, 150, 200, 250 and 300 turns m^{-1} by alternatively flowing 90 °C and 25 °C water. (b) The actuation stroke and the work

capacity as a function of the mass of the applied load for the homochiral $PEHF_{580\text{-}990}$ actuators with different twist densities, which were derived from graphs in (a).

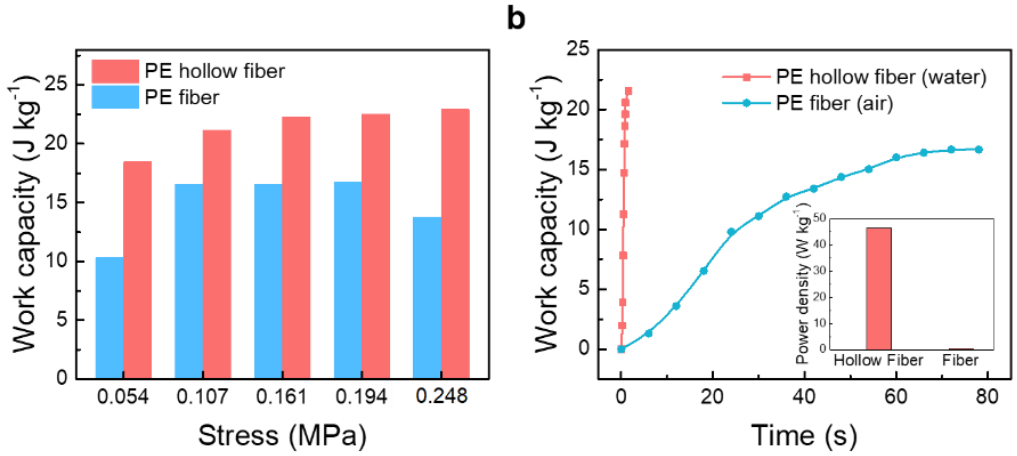

Supplementary Figure 9. (a) Comparison of the work capacity at different isobaric stress for the homochiral $PEHF_{580\text{-}990}$ actuator driven by 90 °C water and for the PE solid fibre actuator driven by 90 °C water. (b) Comparison of the time-dependence of work capacity for the homochiral $PEHF_{580\text{-}990}$ actuator driven by 90 °C water and for the PE solid fibre driven by 90 °C air at the stress of 107 kPa, and the inset was the comparison of power density. The twist density was 300 turns m^{-1}, and the spring index was 4.0. The isobaric stress was calculated as the weight of the load divided by the cross-sectional area of sheath of the hollow fibre.

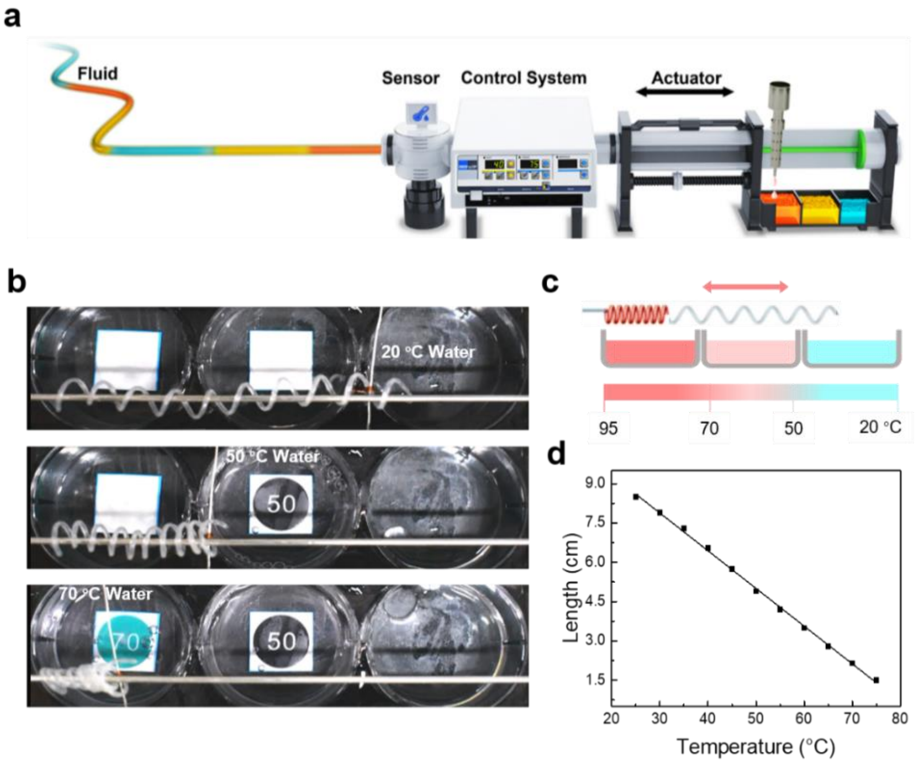

Supplementary Figure 10. (a) Schematic diagram of a conventional microfluidic manipulation system containing liquid sensing, transporting and actuating devices. Optical images (b) and schematic illustration (c) of the contractile hollow fibre actuators used for sensing the liquid temperature and sorting the liquid into the

desired vessels. (d) The length of the hollow fibre actuators as a function of the temperature of the flowing water for the homochiral $PEHF_{580\text{-}990}$ actuators.

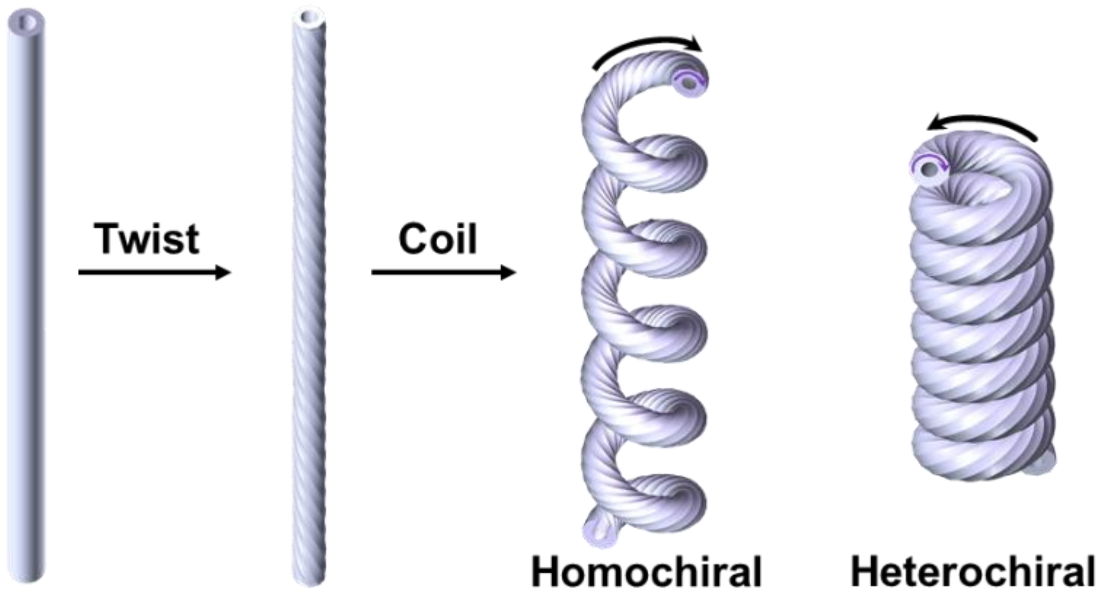

Supplementary Figure. 11 Schematic of the twisted and coiled hollow-fibre actuators

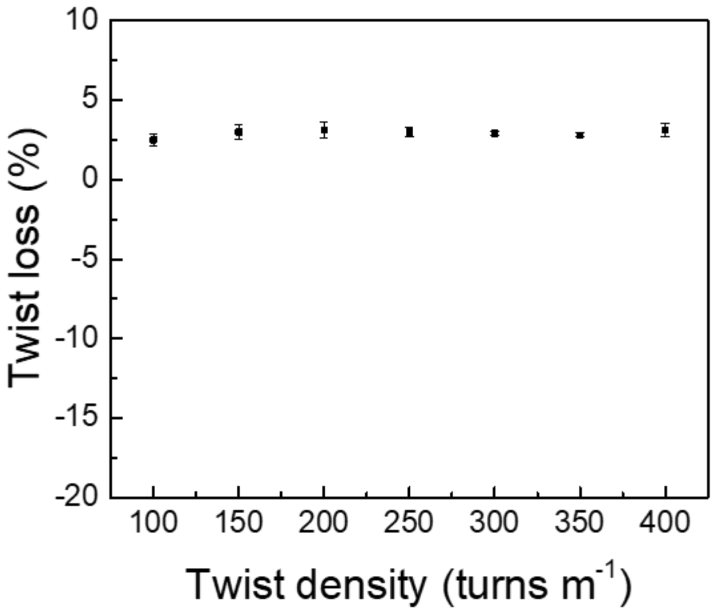

Supplementary Figure 12. The twist loss of the hollow fibre after twist insertion, thermal annealing with both-end tethered at 108 °C for 1h, and cooling down to room temperature and removing the tethering.

Supplementary Tables:

Supplementary Table 1. Response time and actuation parameters of the torsional PEHF actuators in this work.

Materials	Twist density (turns m^{-1})	$\Delta\theta$ (°C)	Flowrate (g s^{-1})	Response time (s)
$PEHF_{580\text{-}990}$	100	70	0.5	2.5
$PEHF_{580\text{-}990}$	200	70	0.5	2.1
$PEHF_{580\text{-}990}$	300	70	0.5	1.9
$PEHF_{580\text{-}990}$	400	70	0.5	1.7
$PEHF_{580\text{-}990}$	400	55	0.5	1.8
$PEHF_{580\text{-}990}$	400	45	0.366	2.7
$PEHF_{580\text{-}990}$	400	35	0.327	1.1
$PEHF_{580\text{-}990}$	400	5	1.72	1.0

Supplementary Table 2. Response time and actuation parameters of the tensile PEHF actuators in this work.

Materials	Twist density (turns m^{-1})	$\Delta\theta$ (°C)	Flowrate (g s^{-1})	Response time (s)
$PEHF_{580\text{-}990}$	200	35	1.72	2.5
$PEHF_{580\text{-}990}$	200	45	1.72	1.6
$PEHF_{580\text{-}990}$	200	55	1.72	1.4
$PEHF_{580\text{-}990}$	200	70	1.72	0.88
$PEHF_{580\text{-}990}$	200	70	0.5	1.4
$PEHF_{580\text{-}990}$	200	70	0.366	1.6
$PEHF_{580\text{-}990}$	200	70	0.327	1.7
$PEHF_{580\text{-}990}$	200	70	0.245	2.8
$PEHF_{280\text{-}640}$	310	70	0.366	1
$PEHF_{380\text{-}1090}$	182	70	0.366	3.0

Supplementary Table 3. Resolution and the coil parameters of the homochiral PEHF hollow fibre actuators in this work.

Fibre Length (l, mm)	Coil Length (L, mm)	Number of Turns (N)	Twist density (T, turns mm^{-1})	Resolution ($l^2\Delta T/NL$, 10^{-3})
200	105	11	0.30	-3.0%
200	105	11	0.25	-2.0%
200	105	11	0.20	-1.3%
200	105	11	0.15	-0.78%
200	105	11	0.10	-0.43%
200	115	12	0.30	-2.5%
200	115	12	0.25	-1.7%
200	115	12	0.20	-1.1%
200	115	12	0.15	-0.66%
200	115	12	0.10	-0.36%
200	130	15	0.25	-1.2%
200	130	15	0.20	-0.78%
200	130	15	0.10	-0.26%
200	145	18	0.20	-0.58%
200	145	18	0.10	-0.19%

References:

1. Gururajan G. & Ogale A. A. Real-time crystalline orientation measurements during low-density polyethylene blown film extrusion using wide-angle X-ray diffraction. *Polym. Eng. Sci.* **52**, 1532-1536 (2012).
2. Dees J. R. & Spruiell J. E. Structure development during melt spinning of linear polyethylene fibers. *J Appl. Polym. Sci.* **18**, 1053-1078 (1974).
3. Choy C., Chen F. & Young K. Negative thermal expansion in oriented crystalline polymers. *J. Polym. Sci. Polym. Phys. Ed.,* **19**, 335-352 (1981).
4. Stein R. S. The X-ray diffraction, birefringence, and infrared dichroism of stretched polyethylene. II. Generalized uniaxial crystal orientation. *J Polym. Sci.* **31**, 327-334 (1958).
5. Jiang P., Li X., Xu R. & Zhang F. Heat extraction of novel underground well pattern systems for geothermal energy exploitation. Renew. Energ. 90, 83-94 (2016).
6. Olga 7 user manual. (2010).
7. Allan J., Pinder H. & Dehouche Z. Enhancing the thermal conductivity of ethylene-vinyl acetate (EVA) in a photovoltaic thermal collector. AIP Advances 6, 035011 (2016).
8. Zhang Z. & Lafleur P. G. Investigation of LDPE blown film instability induced by air cooling. Polym. Eng. Sci. 48, 1504-1510 (2008).
9. Hahn D. W. & Özisik M. N. Heat conduction. John Wiley & Sons (2012).
10. Haines C. S., et al. Artificial Muscles from Fishing Line and Sewing Thread. Science 343, 868-872 (2014).
11. Aziz S, Naficy S, Foroughi J, Brown H. R. & Spinks G. M. Controlled and scalable torsional actuation of twisted nylon 6 fiber. J. Polym. Sci., Part B: Polym. Phys. 54, 1278-1286 (2016).
12. Leng X, et al. Tuning the reversibility of hair artificial muscles by disulfide cross-linking for sensors, switches, and soft robotics. Mater. Horiz. 8, 1538-1546 (2021).
13. Budynas R. G. & Nisbett J. K., Shigley's Mechanical Engineering Design (McGraw-Hill, New York, 2008).
14. Kong J., Fan X., Xie Y. & Qiao W. Study on molecular chain heterogeneity of linear low-density polyethylene by cross-fractionation of temperature rising elution fractionation and successive self-nucleation/annealing thermal fractionation. J. Appl. Polym. Sci. 94, 1710-1718 (2004).
