# Supplementary material for: Microfluidic manipulation by spiral hollow-fibre actuators
Source: Nat Commun. 2022 Mar 14;13:1331. doi: 10.1038/s41467-022-29088-9 (PMC8921237; doi:10.1038/s41467-022-29088-9)
Supplement: Supplementary file 3 — Description of Additional Supplementary Files [file 41467_2022_29088_MOESM3_ESM.docx]

**Description of Additional Supplementary Files**

**File Name: Supplementary Movie 1**.

Description: A homochiral PEHF_580-990_ actuator lifted a 10 g weight and contracted by 50% in 1.2 s, which was recorded by a video camera (Nikon D7500) and a thermal camera (FLIR T440). The twist density was 300 turns m^-1^ and the spring index was 4.0. The maximum surface temperature of the PEHF_580-990_ actuator was recorded by flowing 90 °C water at a flowrate of 1.72 g s^-1^.

**File Name: Supplementary Movie 2**.

Description: An animated video showing a microfluidic manipulation device that can sense the fluid temperature and sort the fluid into the desired vessels for chemical reaction employing a tensile hollow fiber actuator.

**File Name: Supplementary Movie 3.**

Description: A torsional actuator sensing water under different temperatures and sorting water into different vessels. The torsional microfluidic manipulation device was made of a twisted PEHF_580-990_ actuator with a twist density of 400 turns m^-1^. The outlet of the twisted PEHF_580-990_ was made to the “L-shape”. The twisted hollow fiber rotated to different angles upon flowing water with different temperatures.

**File Name: Supplementary Movie 4**.

Description: The translational manipulation device was made of homochiral PEHF_580-990_ actuator, with the twist density of 250 turns m^-1^ and spring index of 5.0. Three 3.6-cm-diameter petri-dishes were placed on the bench side-by-side. The temperature-sensitive tags were sticked in two dishes, which can indicate the water temperature by changing the color from white to black at 50 °C and then to green at 70 °C. The coiled hollow fiber contracted to different lengthes upon flowing water with different temperatures.

**File Name: Supplementary Movie 5**.

Description: The soft clamp was made of the homochiral PEHF_580-990_ actuator with twist density of 250 turns m^-1^ and the spring index of 3.5. By flowing 80 °C water at a flowrate of 1.72 g s^-1^, the actuator shrank to trap a 2-g load. The load was then lifted by hand. Stopping the water flow resulted in temperature decrease of the actuator by heat dissipation to the air, and the load was released.
